# Volasertib preclinical activity in high-risk hepatoblastoma

**DOI:** 10.18632/oncotarget.27237

**Published:** 2019-11-05

**Authors:** Dina Kats, Cora A. Ricker, Noah E. Berlow, Bénédicte Noblet, Delphine Nicolle, Katell Mevel, Sophie Branchereau, Jean-Gabriel Judde, Cody D. Stiverson, Christina L. Stiverson, Matthew N. Svalina, Teagan Settelmeyer, Kevin Matlock, Melvin Lathara, Charlotte Mussini, James I. Geller, Christopher Noakes, Ido Sloma, Narendra Bharathy, Stefano Cairo, Charles Keller

**Affiliations:** ^1^Children’s Cancer Therapy Development Institute, Beaverton, OR, USA; ^2^Research and Development Unit, XenTech, Evry, France; ^3^Bicêtre Hospital, Le Kremlin Bicêtre, France; ^4^Omics Data Automation, Beaverton, OR, USA; ^5^Division of Oncology, Cincinnati Children’s Hospital Medical Center, Cincinnati, OH, USA; ^6^Champions Oncology, Rockville, MD, USA

**Keywords:** hepatoblastoma, PLK1, volasertib, liver, cell cycle

## Abstract

Relapsed and metastatic hepatoblastoma represents an unmet clinical need with limited chemotherapy treatment options. In a chemical screen, we identified volasertib as an agent with *in vitro* activity, inhibiting hepatoblastoma cell growth while sparing normal hepatocytes. Volasertib targets PLK1 and prevents the progression of mitosis, resulting in eventual cell death. PLK1 is overexpressed in hepatoblastoma biopsies relative to normal liver tissue. As a potential therapeutic strategy, we tested the combination of volasertib and the relapse-related hepatoblastoma chemotherapeutic irinotecan. We found both *in vitro* and *in vivo* efficacy of this combination, which may merit further preclinical investigation and exploration for a clinical trial concept.

## INTRODUCTION

Hepatoblastoma is the most common primary liver tumor diagnosed in children, with approximately 150 cases per year in the United States. Hepatoblastoma incidence has risen by about 4% between 1992 and 2004, more so than any other childhood cancer [1]. While the survival rate for patients whose tumor can be surgically removed approximates 90% [2] when supported by neoadjuvant and/or adjuvant chemotherapy the options for children with unresectable or metastatic disease are limited, often necessitating liver transplantation and/or aggressive chemotherapy to provide a chance for cure. Thus, the lack of new agents in use for the treatment of hepatoblastoma is a substantial unmet clinical need. In addressing this issue, scientists and clinicians have pursued biological treatments that target the molecular mechanisms of hepatoblastoma proliferation and metastasis. To date, several targets have been identified for hepatoblastoma, including NK1R [3], EPCAM [4, 5], LRH-1 [6] and PIM3 [7]. Here, we further identify polo-like kinase 1 (PLK1) as a potential therapeutic target in hepatoblastoma that may have a favorable therapeutic index.

The PLK family of kinases consists of five mammalian homologs, PLK1 to PLK5, with each homolog having a largely non-redundant function in the cell cycle [8]. PLK1 is the most well studied of these five proteins and controls important steps in the transition from G_2_ to mitosis [8]. PLK1, mRNA, and protein expression changes dynamically with the cell-cycle, with peak levels in late G_2_ and M phase [9]. PLK1, PLK2, PLK3, and PLK4 have roles in the G1-S phase transition and centriole duplication, while PLK5 does not have kinase activity [8]. Consistent with PLK1’s biological role in cell cycle regulation, PLK1 overexpression has been observed in a variety of cancer types, including melanoma, breast, non–small cell lung (NSCLC), colorectal, prostate, pancreatic, ovarian, and head and neck cancers, as well as non-Hodgkin lymphoma (NHL) and acute myeloid leukemia (AML) [10]. In pediatric cancer, PLK1 is over-expressed 2.6 fold in childhood acute lymphoblastic leukemia [11] and 1.5 fold in rhabdomyosarcoma, and is highly expressed in pediatric Ewing sarcoma, neuroblastoma, and osteosarcoma [12].

In 2004, Yamada *et al* [13] performed expression profiling of 74 hepatoblastoma samples and compared them to their matched normal tissue. The authors found that the only over-expressed oncogene was PLK1 [13]. Despite evidence of PLK1 over-expression, PLK1 inhibitors have not been pre-clinically or clinically tested for hepatoblastoma.

Volasertib belongs to the dihydropteridinone class of compounds and works by competitively binding to the ATP site in the PLK1 [14, 15]. Volasertib binds to PLK1, PLK2 and PLK3, but has a modest selectivity for PLK1 (cell-free enzyme IC_50_ values of 0.87, 5, and 56 nM for PLK1, PLK2, and PLK3, respectively) [16]. Volasertib has been used in both Phase I and Phase II clinical studies, including for pediatric AML (NCT01971476), but has not been investigated for hepatoblastoma. Clinical trials in other solid tumors have shown that volasertib monotherapy may have limited benefits, but volasertib can be combined with chemotherapy for additive or synergistic effect [17]. A current chemotherapy used for relapsed hepatoblastoma is irinotecan [18]. In this study we show efficacy of volasertib and irinotecan for hepatoblastoma *in vitro* and suggest possible combined efficacy *in vivo*.

## RESULTS

### Volasertib is identified as an agent with a high therapeutic index for hepatoblastoma versus hepatocytes

**Figure 1:**
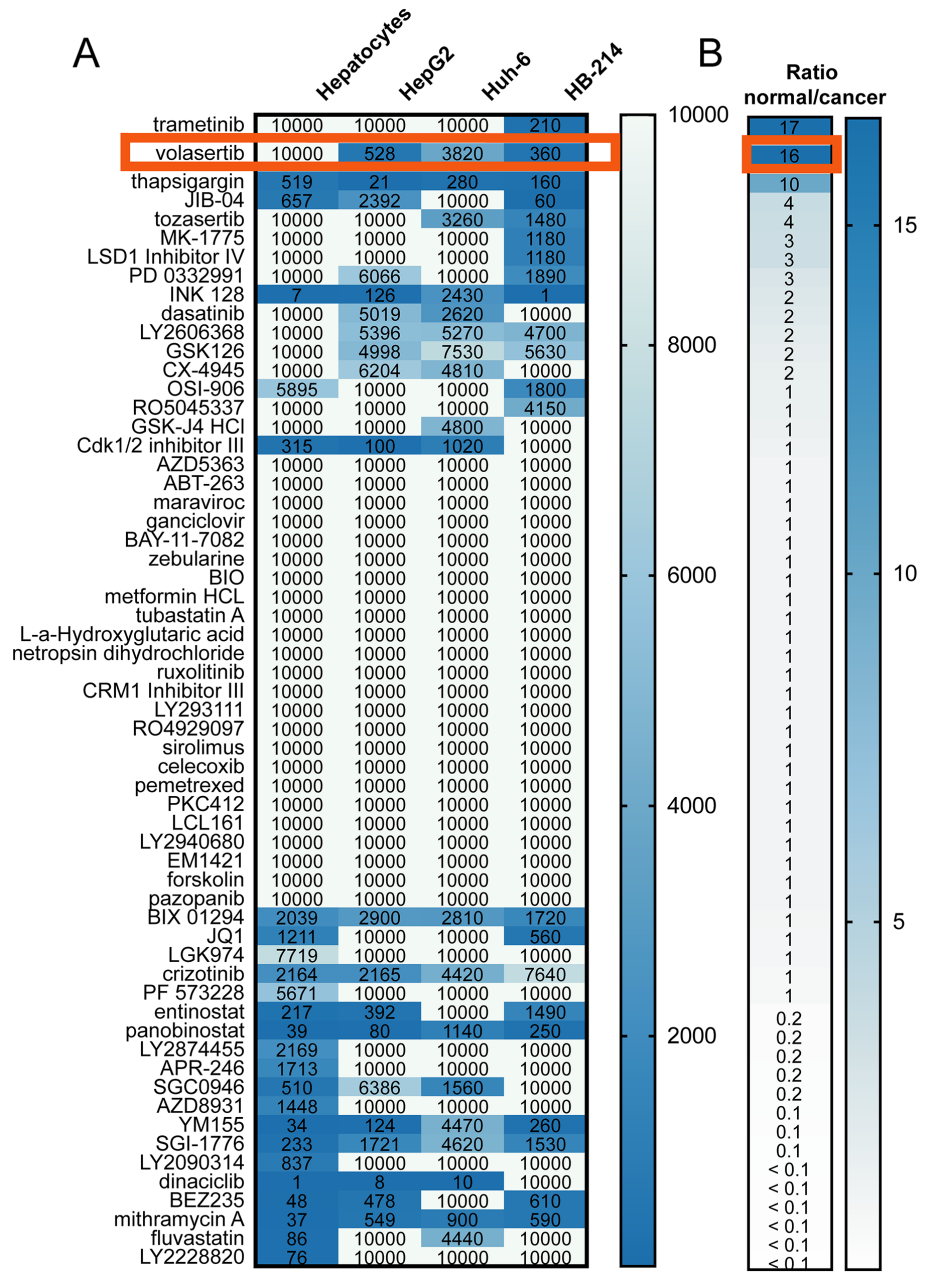
A 60-agent chemical screen of hepatoblastoma cell lines and hepatocytes identifies an **(A)** absolute IC50 (nM) value for each cell line and drug. A value of 10,000 indicates that the drug did not inhibit viability. **(B)** Average ratio of the IC50 in hepatocytes to the IC50 in hepatoblastoma cell lines.

To address an unmet clinical need for new targeted agents for the treatment of hepatoblastoma, we performed a 60-agent chemical screen with a cell viability endpoint using three hepatoblastoma cell lines, HepG2, Huh-6 and HB-214 (a cell line derived from a hepatoblastoma patient derived xenograft (PDX)) [19]. These 60 drugs were chosen based on drugs currently in clinical trials as well as literature about potential drug targets in pediatric cancer. This screen was not intended to be exhaustive, but rather a carefully curated one of targets of contemporary interest or clinical investigation and other drug candidates of opportunity. We identified 10 drugs with an IC50 below 10 μM in all three cell lines (Figure 1A). To refine hits, we also tested normal human hepatocytes to identify drugs that inhibit cancer cell growth, but not normal hepatocytes (Figure 1B). Thapsigargin, volasertib, and trametinib met this criterion. However, trametinib was only effective in one cell line out of three, and thapsigargin is known to have significant renal toxicity [20]. Volasertib was therefore chosen for continued study.

### PLK1 is selectively over-expressed in hepatoblastoma

**Figure 2:**
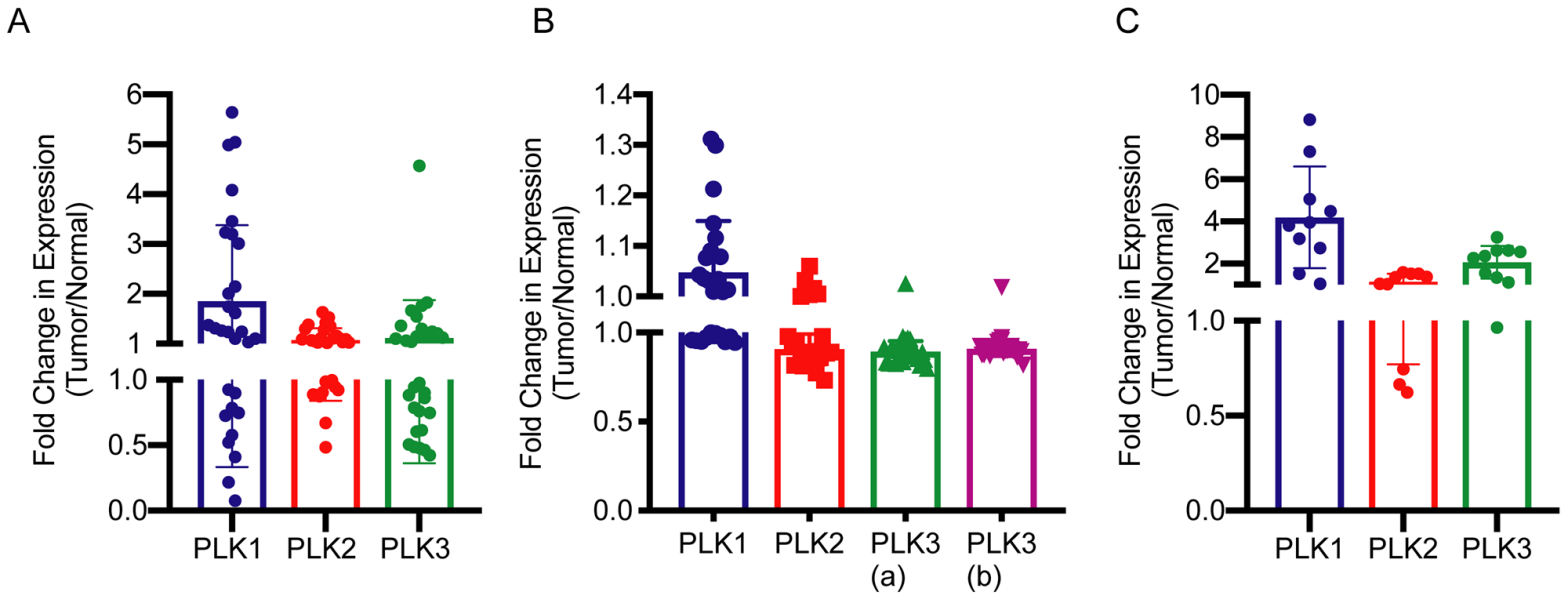
Fold change in mRNA expression of PLK1, PLK2 and PLK3 in hepatoblastoma as compared to normal liver tissue **(A)** mRNA expression data from Hooks, *et al* [21]. Fold change was found to be statistically significant from a hypothetical value of 1 by student’s *t*-test, p = 0.0045. **(B)** Microarray mRNA expression data from Cairo, *et al* [22]. Fold change was found to be statistically significantly different from a hypothetical value of 1 by student’s *t*-test, p = 0.03. PLK3 a, b refer to different Affymetrix probes. **(C)** mRNA expression data from Ranganathan, *et al* [23]. Fold change was found to be statistically significantly different from a hypothetical value of 1 by student’s *t*-test, p =0.0024.

To validate volasertib’s selectivity for hepatoblastoma cells over normal hepatocytes we investigated the expression of PLK1 in normal and hepatoblastoma tumor tissue by using publically available genomic databases [21, 22]. In one study, RNA was sequenced from tumor (n = 25) and matched normal liver tissues (n=25) [21]. In another study, RNA expression was analyzed by microarray from tumor (n=25) and normal (n=4) [22]. A third study performed RNA sequencing for tumor (n=10) and normal (n=3) [23]. In all three sets of data, PLK1 was overexpressed (absolute mean expression 1.51 TPM ± 0.25 SEM, 6.49±0.13 and 1.95±0.36, respectively) with 1.9±0.28 average fold change between tumor and normal (Figure 2A) from the first study, with 1.05± 0.02 fold change (Figure 2B) from the second study and 4.19±0.08 fold change from the third study (Figure 2C). Fold change was statistically significantly different for all three (Student’s *t*-test p<0.05).

### Hepatoblastoma cell line development and characterization

To introduce additional contemporary independent biological replicates, we sequenced and analyzed over 40 million paired-end RNA-seq reads from six hepatoblastoma PDX-derived cell culture generated at XenTech, which were validated continuously using STR analysis (Supplementary Table 1). Clinical information for these cell cultures is given in Supplementary Table 2. All cell cultures were found to secrete alpha-fetoprotein, a defining characteristic of hepatoblastoma which is not present in fully differentiated, non-regenerating hepatocytes. (Supplementary Figure 1). The cell lines have an average doubling time of 70 hours, as compared to 48 hours for HepG2. The recent derivation of these cell lines from PDX samples makes these cultures potentially more representative of hepatoblastoma than the few other commonly used cell lines (Huh-6, HepG2). Specifically, HepG2 was derived from a 15-year-old patient, which is uncharacteristic of hepatoblastoma; however, HepG2 has been determined in the literature to truly be hepatoblastoma and not hepatocellular carcinoma [24]. While six cell lines may seem limited, there are only 3 publically available commonly used hepatoblastoma cell lines; thus, the addition of six biologically representative samples could be viewed as a technical advance in this field.

**Figure 3:**
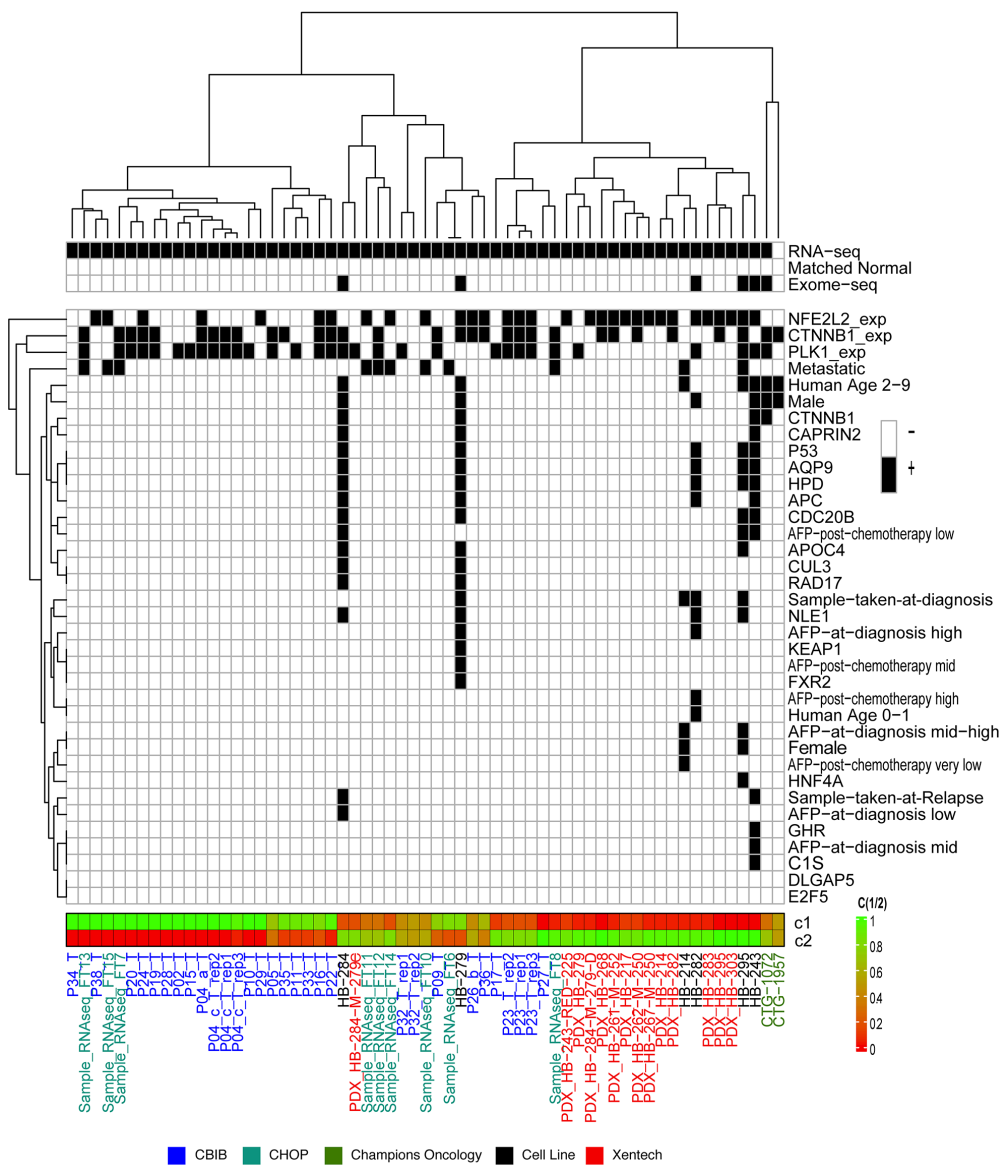
16-Gene signature endotypes Unsupervised clustering of RNA sequencing from hepatoblastoma samples using the pre-defined 16-gene signature^20^. Hepatoblastoma cell lines (black), patient-derived xenograft (PDX) models from Champions Oncology (green), tumor tissue samples from the University of Bodeaux (CBIB, blue), and tumor tissue samples from Children’s Hospital of Philadelphia (CHOP, purple) are clustered into three major groups. Samples that had RNA sequencing, whole-exome sequencing, and/or match normal DNA sequencing are indicated at the top of the legend. Below, samples with genes with somatic mutations, overexpressed genes, and clinical and demographic information are marked by the black box. Unsupervised clustering was performed on the data within the legend (vertical dendrogram). Below the legend, samples are scored on a scale of 0 to 1 to be in either the C1 or C2 groups determined by Cairo, et al [22]. AFP values are indicated as follows: AFP high is in the range of 1,000,000 – 10,000,000, AFP mid-high is between 100,000 and 999,999, AFP mid is between 10,000 and 99,999, AFP mid-low is between 1,000 and 9,999 and AFP low indicates a value between 0 and 999.

To determine how the cell lines are classified among other hepatoblastoma samples, we performed unsupervised clustering analysis using RNA sequencing data from PDX-derived hepatoblastoma cell lines (n=6); patient tissue samples (n=39); publicly available, unpublished PDX models (n=2) from Champions Oncology; and Xentech PDX models (n=14) [21, 23]. We found that 60% (18/30) of samples with high PLK1 expression (above median PLK1 TPM of all tumor samples) also had high β-catenin (CTNNB1) expression (above median CTNNB1 TPM of all tumor samples) (Figure 3). We used the 16 gene signature described by Cairo, *et al* [22] to distinguish these samples into the C1 or C2 molecular phenotype [22]. C2 classification has been shown to be correlated with a poor prognosis [22]. Of the 60 samples tested, 30 showed a C2-like profile, including five out of the six cell lines. The cell lines classifying into the C2 category may be mostly or purely related to their rapid growth phase as compared to tumor tissue. However, this finding may be indicative that gene expression in the cell lines reflects the “biological state” of more aggressive clinical samples. Twenty-six out of the 30 C2 categorized samples also expressed high PLK1, and 3 out of the 29 C1 categorized samples expressed high PLK1. Differential expression analysis was performed on metastatic vs primary tumor samples utilizing a quasi-likelihood test on a Genewise Negative Binomial Generalized Linear Model utilizing *EdgeR* [25]. From this analysis we uncovered that the PLK1 expression from primary samples was found to be higher than metastatic samples (2.37 log fold change p = 0.018). In addition, we found that of the 9 samples from metastatic cancer, 3 had high PLK1 (higher than the median).

To cross validate the overexpression of PLK1 in aggressive hepatoblastoma, we used the 16-gene classifier on another separate set of microarray data from 55 hepatoblastoma samples [26]. In the microarray series, samples were separated into two main cluters. The cluster with C2 phenotype was associated with aggressive clinical feature and high PLK1 expression (Supplementary Figure 2), notably with PLK1 showing high positive correlation with DLG7 (Pearson correlation R=0.4715, p = 0.0279) and BUB1 (R=0.3917, p = 0.00313), two genes strongly involved in the mitotic checkpoint. PLK1 overexpression in hepatoblastoma has been previously characterized [13] as a potential therapeutic target and here we show that PLK1 is also overexpressed in our sample set. We also performed unsupervised clustering analysis using both RNA and DNA sequencing, and included more genes known to be associated with hepatoblastoma progression (Supplementary Figure 3).

### Volasertib and SN38 block hepatoblastoma cell proliferation at clinically relevant concentrations

**Figure 4:**
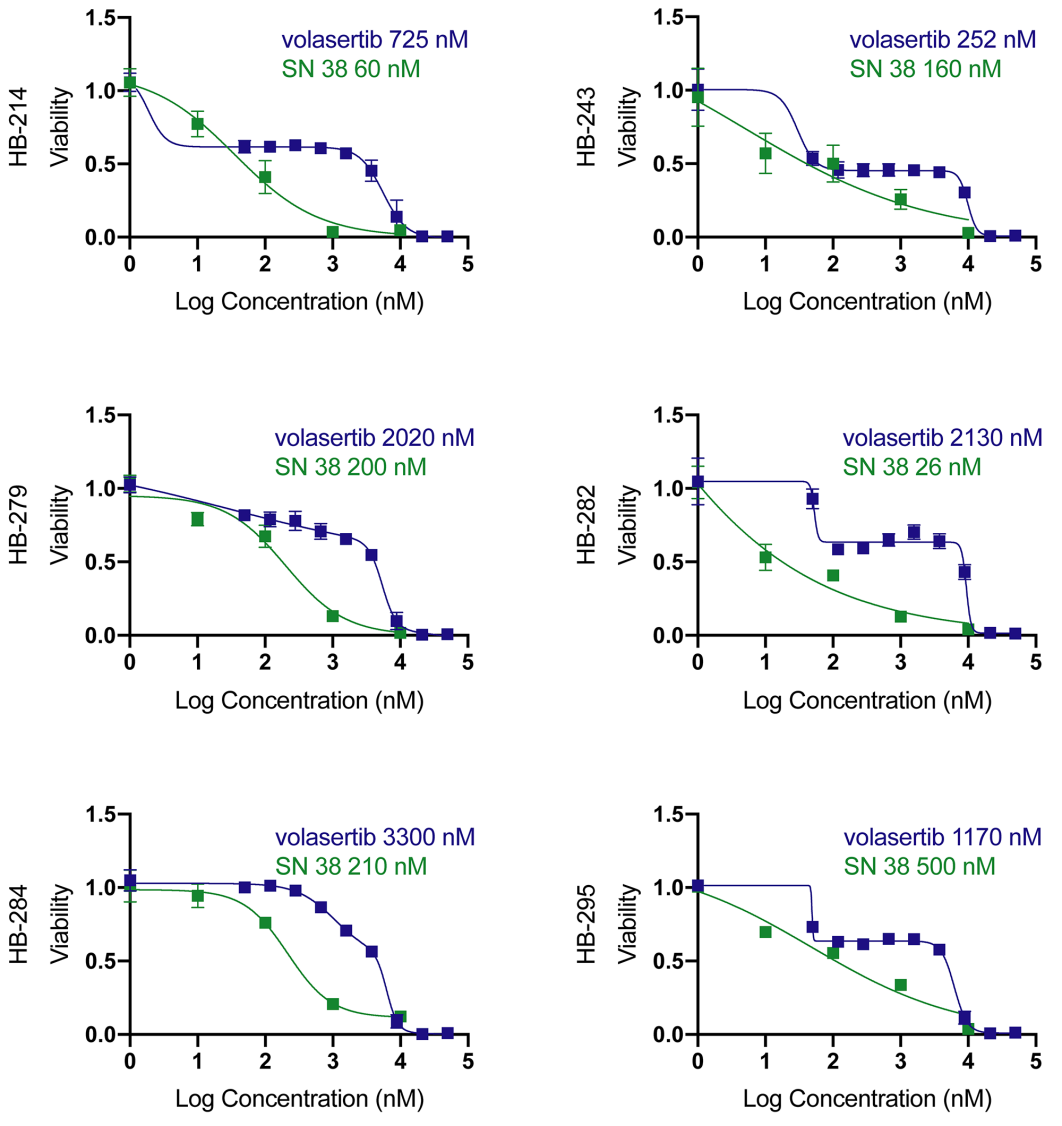
Volasertib and SN38 have clinically relevant absolute IC50s Volasertib IC50s range from below to slightly above calculated Cmax (1220nM [32]). SN38 IC50s are clinically relevant. Values are an average of quadruplicates, Data is represented as mean+/− standard deviation.

**Figure 5:**
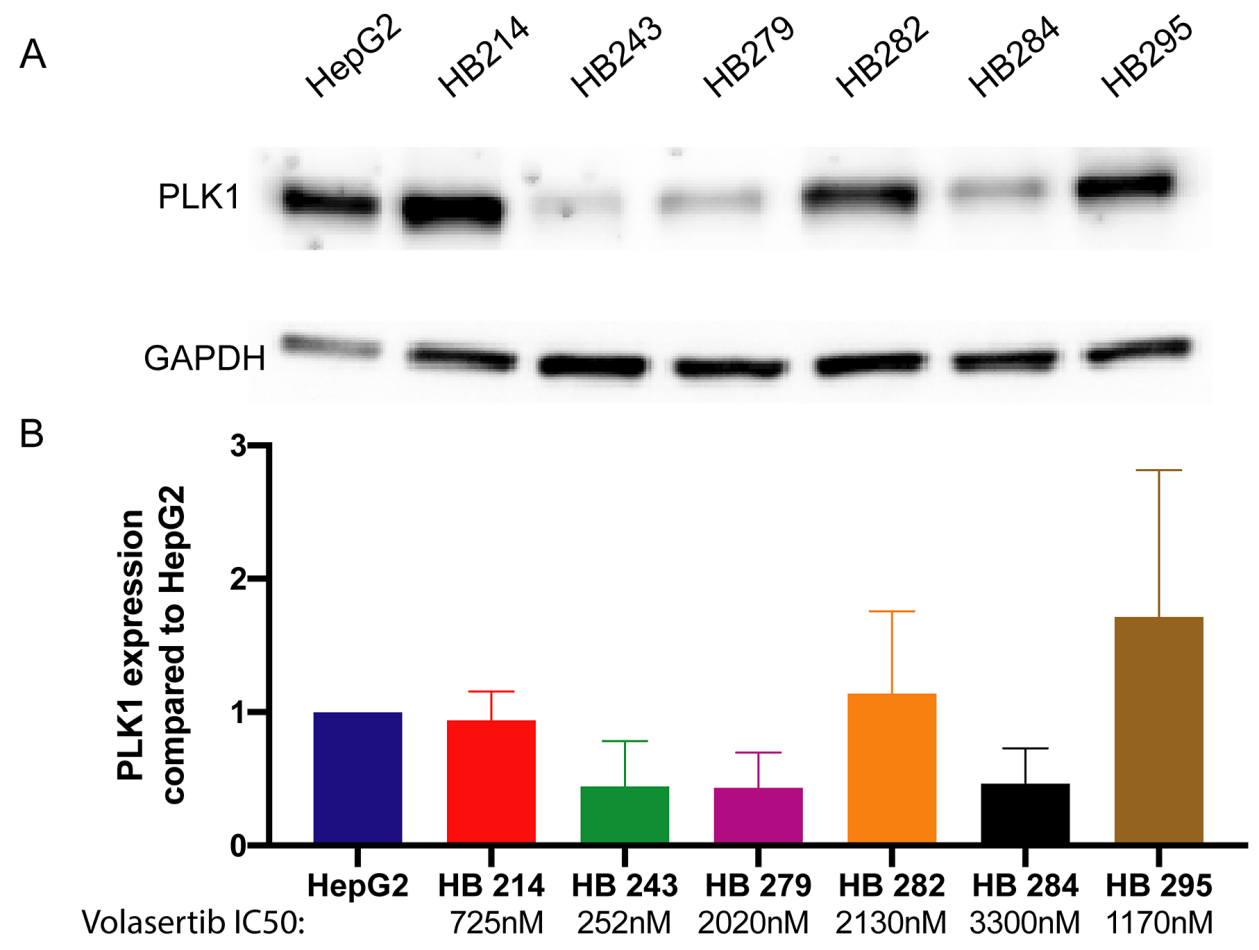
Hepatoblastoma cell lines express varied amounts of PLK1 **(A)** Hepatoblastoma cell lines were measured for PLK1 protein expression by western blot. **(B)** PLK1 expression levels in each cell line were quantified by western blot and compared to HepG2 PLK1 expression levels.

We tested volasertib in our six new hepatoblastoma cell cultures. All cultures had an IC50 for volasertib below 10,000 nM, with a range between 252 nM and 3500 nM (Figure 4). This range is in contrast with IC50 values calculated for volasertib in hepatocellular carcinoma cell lines, which were 3970 nM and 7025 nM for SMMC7721 and SK-Hep1, respectively [27]. The wide range of hepatoblastoma IC50 values may be a function of the longer doubling time of hepatoblastoma cells. Almost all cell lines had a biphasic response to volasertib, which is consistent with some other publications and suggests that at clinically achievable doses volasertib is cytostatic and at higher doses volasertib is cytotoxic [28]. From available clinical trial data (pediatric and adult), the C_max_ ranged from 300 nM to 1700 nM, depending on dosage and interval [29–33]. One half of cell cultures had an IC50 below 1700 nM. We further verified the presence of the target, PLK1 in the cell lines by western blot. Half of the cell lines had relatively high PLK1 expression (Figure 5); however, PLK1 expression did not correlate with volasertib sensitivity as evidenced by a Spearman correlation coefficient of 0.25, consistent with other publications where PLK1 mRNA level did not correlate with volasertib resistance [34].

Since half of the cell cultures responded under the published maximum serum concentration (C_max_) for volasertib, we chose to test whether hepatoblastoma cell cultures would also be sensitive to SN38, the active irinotecan metabolite and a potential combination agent. Irinotecan is a chemotherapeutic currently used for cases of relapsed hepatoblastoma [18]. All cell cultures had an IC50 of between 26 and 500 nM for SN38 (Figure 4). The C_max_ for SN38 from clinical trials was between 30nM and 120 nM [30, 35]. HB-214 showed the greatest sensitivity to SN38 with an IC50 of 60 nM (Figure 4). Vincristine is also commonly used for relapsed hepatoblastoma [36], but only one cell line had an IC50 below the published C_max_ of 90 nM [37]. Several cell lines were highly resistant to vincristine, with one cell line being completely resistant (Supplementary Figure 4).

### Volasertib has synergy with SN38 *in vitro*

**Figure 6:**
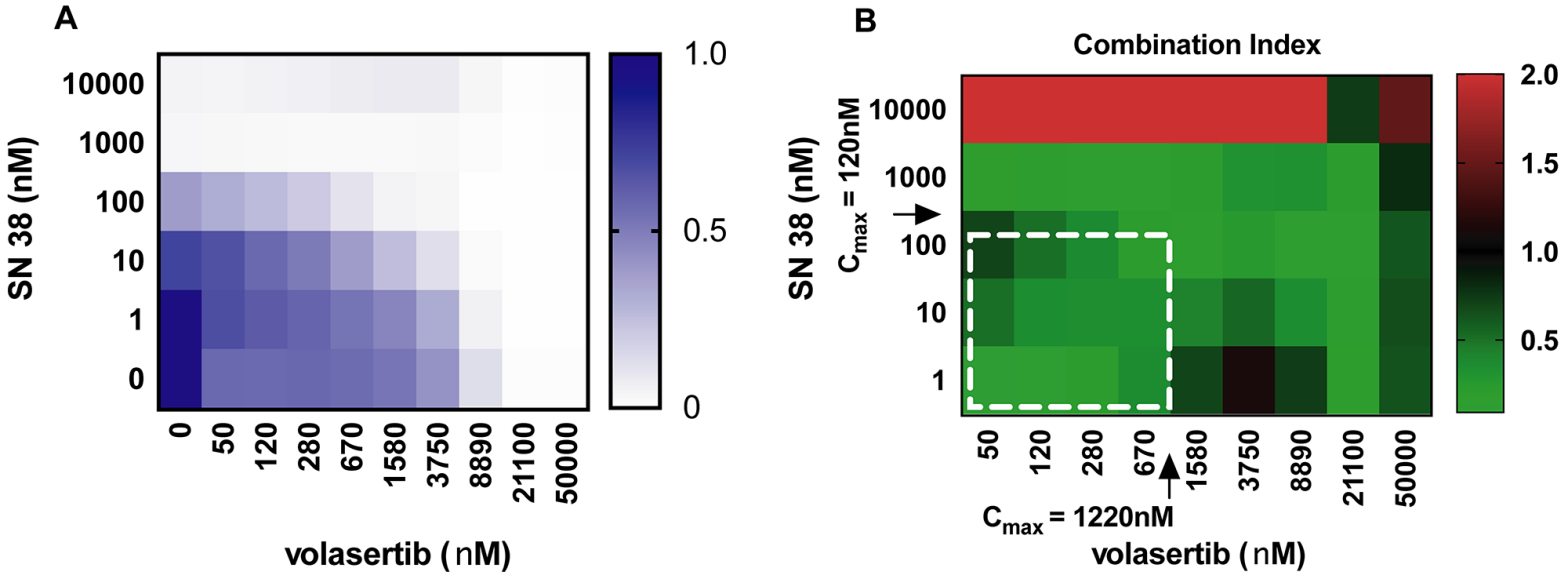
Heatmap of drug response in HB-214 **(A)** Cell proliferation in response to 72-hour drug treatment. **(B)** Combination index of volasertib and SN38 drug treatments. White dotted line denotes clinically achievable concentration ranges. N = 4.

Hepatoblastoma cell cultures were treated with varying concentrations of both volasertib and SN38 for 72 hours and cell growth was assessed. Combination index (CI) values were calculated for each drug combination. Combination index is a standard measure of combination effect that indicates a greater (CI<1), lesser (CI>1) or similar (CI=1) effect than the expected additive effect. Synergy (defined as a combination index value less than one) existed for each cell line at concentrations below the Cmax of each drug, including for cell lines that were more resistant to SN38 (Figure 6, Supplementary Figure 5). By contrast, combinations with vincristine had antagonism (CI>1) at most concentration used (Supplementary Figure 6).

### Combined SN38 and volasertib causes both S or G_2_/M cell cycle arrest

**Figure 7:**
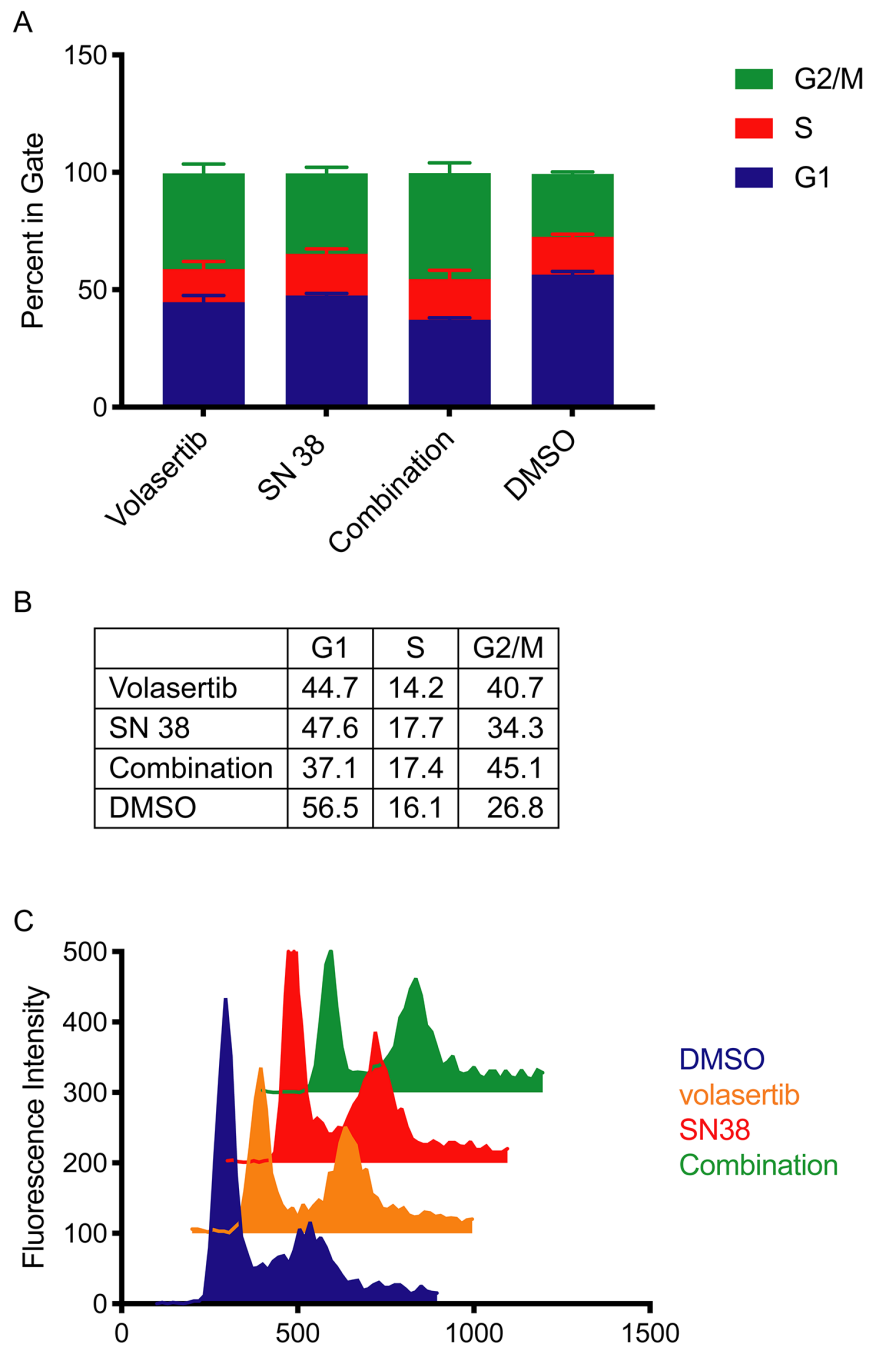
Volasertib causes cell cycle arrest in G2/M after 72 hours treatment HB-279 was treated with volasertib for 72 hours and cell cycle was analyzed by PI staining and flow cytometry. N=3, data is represented as mean +/− SD. Percent of cells in G1, G2/M and S phase after 72 hour treatment with volasertib, SN38, the combination of volasertib and SN38 or DMSO represented as a **(A)** bar graph and **(B)** numerical values. **(C)** Representative histograms of cell cycle distribution after drug treatment.

Volasertib and SN38 are both known to cause disruptions in the cell cycle. Volasertib causes arrest in early M phase and results in a monopolar spindle, halting entry into prometaphase. Volasertib-induced cell-cycle arrest has been shown across several model systems [15, 38, 39] and is consistent in a representative hepatoblastoma cell culture (Figure 7A). SN38 inhibits topoisomerase I activity by binding TOP1 and DNA, preventing re-ligation of the DNA and causing arrest in S phase. Once the cleavable TOP1-DNA complex collides with replication or transcription machinery, single-strand DNA breaks that resulted from TOP1 activity become double-stranded breaks and cause G_2_ phase arrest and eventual cell death; this result holds true across multiple model systems [40–42] and a representative hepatoblastoma cell culture (Figure 7A). To test for synergy between these interventions, we exposed hepatoblastoma cell lines to clinically relevant dosages of SN38 and volasertib. Concentrations of drug were determined by estimating clinically used area under the curve (AUC) values, and dividing by 72 hours to achieve a similar cumulative drug exposure *in vitro*. The published AUC_0-∞_ value of volasertib in pediatrics is between 8000 to 13000 nM^*^h [29, 31–33], which is comparable to 150 nM volasertib over 72 hours. The published AUC_0-∞_ value of SN38 from a pediatric study was 214 nM*h [35], which corresponds to an *in vitro* concentration of 3 nM for 72 hours. When exposing hepatoblastoma cell cultures to these clinically achievable concentrations of drug which were also used in our cytotoxicity studies, we observed cell cycle arrest in G_2_/M (4N ploidy) for volasertib treatment and a modest G_2_/M phase arrest for SN38 treatment as compared to vehicle control (Figure 7). We then observed a statistically significant decrease in the S phase and an increase in the G_2_/M phase for SN38 alone and the drug combination (p=0.0004, p<0.0001 respectively) by two-way ANOVA (Figure 7).

### Volasertib combined with SN38 has efficacy *in vivo* in hepatoblastoma PDX

**Figure 8:**
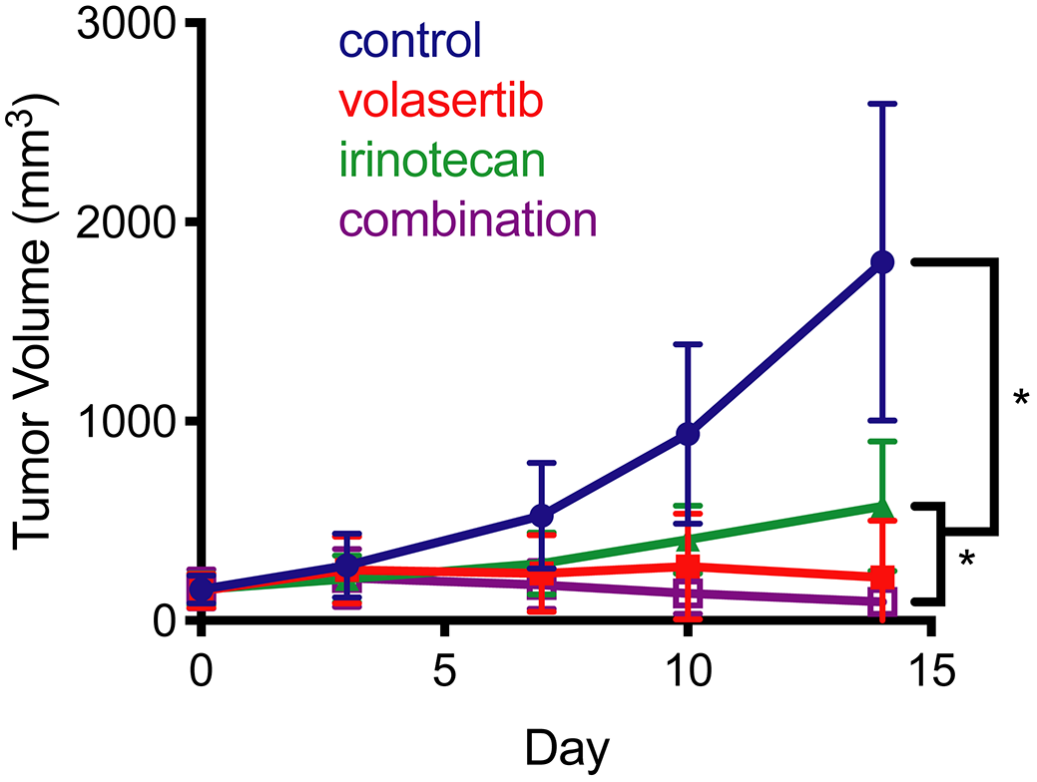
Xenograft studies of irinotecan and volasertib Tumor growth was slowed when treated with volasertib, irinotecan or the combination. Mean tumor volume over time. ^*^ indicates p < 0.004 by the Mann-Whitney test.

To test whether our *in vitro* results translated *in vivo*, we treated PDX mice bearing human hepatoblastoma tumors with volasertib and irinotecan as single agents or a combination of both drugs. The study design is given in Supplementary Table 3. We selected HB-214 PDX because the resultant cell line had the greatest sensitivity to volasertib and SN38 in our *in vitro* studies. A statistically significant difference in tumor size at day 14 was found between the control group and each treatment group (Figure 8), although the effects of the combination were modest. However, single agent activity for volasertib at the dose tested was appreciable and cytostatic.

## DISCUSSION

At the time of diagnosis, between one-third to two-thirds of hepatoblastoma patients do not have a resectable tumor and must be treated with chemotherapy to make surgery feasible [43]. Chemotherapeutics that have been commonly used include cisplatin, doxorubicin, carboplatin and irinotecan. Clinically, the empirically driven advancements in surgery have improved the outcomes for hepatoblastoma. Surgical strategies include conventional surgical resection or liver transplant. Salvage chemotherapeutic strategies do not work for every patient, and can result in long-term side effects, including severe hearing loss [44], infertility [45] and thinning of the heart wall [46].

To address this need, we have explored the activity of volasertib, which had selectivity for cancer cells (IC50 360-528 nM) compared to normal hepatocytes (IC50 > 10 μM) in cell lines derived from hepatoblastoma PDXs. This dose is clinically achievable (C_max_ measured in clinical trials is 1220 nM [32]). These hepatoblastoma cell lines were developed from aggressive hepatoblastoma cases as indicated by the clinical history of the patients (Supplementary Table 2), and are therefore representative of poor prognosis hepatoblastoma and serve as a tool to identify second line treatments.

Volasertib has been investigated clinically in pediatric leukemia but has not yet been studied in hepatoblastoma. The mechanism of volasertib and its specificity for PLK1 and interaction with BRD4 has been studied extensively [16]. For other cancers, volasertib-chemotherapy combinations are being explored in lieu of volasertib monotherapy [17]. To that end, irinotecan is a chemotherapy agent for hepatoblastoma (*i.e.*, NCT00980460) although irinotecan has an overlapping toxicity profile with volasertib. Irinotecan causes diarrhea and neutropenia [30, 35], and the side effects of volasertib are neutropenia and anemia [6, 47]. The drug biologies are not completely unlinked: a previous study showed that camptothecin sensitivity (including irinotecan) is determined by PLK1 expression in squamous cell carcinoma, and that combined treatment of irinotecan and the PLK1 inhibitor BI2536 increased the antitumor effect of irinotecan [48]. Volasertib is a derivative of BI2536 generated to create a more potent PLK1 inhibitor [17].

In the present study, we show tumor growth inhibition when volasertib and irinotecan are combined in a (single) PDX model of hepatoblastoma. Comparative studies of human and rodent clearance suggest a lower dose of volasertib may be appropriate in preclinical studies [16]. Therefore, we propose that volasertib in combination with irinotecan may be a viable concept for recurrent and/or metastatic hepatoblastoma worthy of additional preclinical investigation. Furthermore, *in vivo* testing with several PDX models for a treatment period longer than 30 days and with close attention paid to clinically achievable dosages is warranted.

In this study we used the most sensitive cell line for *in vivo* testing, and we note that a wider range of cell lines would be beneficial, especially because some cell lines had IC50s for volasertib exceeding 1µM. XenTech has developed an additional 24 unpublished hepatoblastoma PDXs and such studies are planned as a future direction. Further studies could include the analysis of PLK1 expression/phosphorylation status in other high-risk hepatoblastoma histology cohorts to determine the possible benefit of volasertib treatment for other groups. Nevertheless, the results presented here provide a foundation for these future studies.

## MATERIALS AND METHODS

### Cell lines

HepG2 was purchased from ATCC (HB-8064, ATCC, Manassas, VA) and maintained in DMEM supplemented with 10% FBS and 1% penicillin-streptomycin. Huh-6 was received from the RIKEN (Japan) cell bank and maintained in DMEM with 10% FBS. HB-214, HB-295, HB-282, HB-284, HB-243 and HB-279 (Supplementary Figure 7) were developed at Xentech (Every, France) from hepatoblastoma PDXs and maintained in Advanced DMEM with 8% FBS, 1% L-glutamine, 1% penicillin-streptomycin along with 20 µM Rock Kinase inhibitor. Cell cultures were authenticated by short tandem repeat analysis using the Promega PowerPlex16HS Assay (Madison, WI).

### 60 agent screen

On day 0, cells were trypsinized and plated into a white walled 384-well plate containing pre-diluted known concentrations of various drugs. After 72 hours, an equal volume of Cell-Titer Glo 2.0 (G9243, Promega) was added to each well, incubated at room temperature while rocking in the dark for 15 minutes, followed by luminescence acquisition with a BioTek Synergy HT plate reader (BioTek, Winooski, VT).

### Alpha-fetoprotein ELISA

Hepatoblastoma cell cultures were maintained without changing media for five days at which point media was collected for sampling. Media from a breast cancer cell line (MDA-MD-231) was used as a control. ELISA was performed according to the manufacturer’s protocol (ab108838, Abcam, Eugene, OR).

### Cell proliferation studies

On day 0, cells were trypsinized and plated into white walled 384-well plates. On day 1, when cells were 70% confluent, volasertib (S2235, SelleckChem, Houston, TX), SN38 (S4908, SelleckChem) or vincristine (S1241, SelleckChem) suspended in DMSO were added to plates using a Tecan D300e drug printer (Tecan Life Sciences, Switzerland), ensuring less than 1% DMSO. On day 4, an equal volume of Cell-Titer Glo (G9243, Promega) was added to each well, incubated at room temperature while rocking in the dark for 15 minutes, followed by luminescence acquisition with a BioTek Synergy HT plate reader. Absolute IC50 values were calculated using GraphPad Prism (GraphPad, San Diego, CA). Synergy values were calculated using CalcuSyn (Biosoft, Cambridge, UK) software.

### Western blotting

Cell lysates were collected RIPA buffer (Thermo Fisher) containing protease and phosphatase inhibitors when cells were 70% confluent. Proteins were separated by SDS-PAGE, and transferred to polyvinyldifluoride membranes. Membranes were blocked in 5% milk in tris-buffered saline with tween 20 and incubated with either anti-PLK1 (208G4, Cell Signaling Technology, Danvers, MA) or anti-GAPDH (14C10, Cell Signaling Technology). After incubation with anti-rabbit horseradish peroxidase antibodies (Vector Laboratories, Burlingame, CA), ECL Western Blotting Detection Reagents (Bio-Rad, Hercules, CA) were used to detect the signal on an IVIS Lumina (Perkin Elmer, Waltham, MA).

### Cell cycle analysis by flow cytometry

After a 72-hour drug treatment, cells were collected and fixed in ice cold 70% ethanol. After rehydration, cells were stained using the Guava Cell Cycle Reagent for Flow Cytometer (4500-0220, MilliporeSigma, Germany) according to the product protocol. Cells were analyzed for propidium iodide using the Guava EasyCyte 8HT (MilliporeSigma). Flow data was analyzed using CytoBank software (Cytobank, Santa Clara, CA).

### PDX establishment and *in vivo* studies

The hepatoblastoma PDXs used to derive the cellular models and perform the *in vivo* studies were generated at XenTech. At surgery, tumor fragments were sampled from the resected tumor and placed in cell culture medium supplemented with penicillin/streptomycin with or without 5% bovine serum albumin (BSA) on ice. Tumor samples were chopped into 4x3 mm fragments and grafted in the interscapular region of 6-8 week-old female athymic nude mice (Athymic Nude-Foxn1^nu^, ENVIGO, Gannat, France). Tumor growth from first implantation occurred with a delay spanning between 1 and 5 months. Growing tumors were serially transplanted onto recipient mice and underwent comparative examination to confirm preservation of their histological features. To immortalize each PDX, vials of 4x3 mm fragments from tumors at different passages were placed in a solution of 90% FCS/10% DMSO or glycerol, and stored at -150°C.

The *in vivo* studies were performed at XenTech. From donor mice, established HB-214 PDX were collected to provide tumor fragments. These fragments were engrafted in the interscapular region of 6-8 week-old female athymic nude mice (Athymic NudeFoxn1^nu^, ENVIGO, Gannat, France). After latency period, mice with a subcutaneously growing tumor between 62 and 256 mm^3^ were allocated to each treatment arm according to their tumor volume to obtain homogenous mean and median tumor volume in each arm. Treatments were randomly attributed to boxes housing up to five mice. All treatment protocols were based on previous reports or internal knowledge on treatment toxicity and administration schedule, detailed protocols are available upon request. Tumor volume (TV) was evaluated by caliper measurement, 2 or 3 times a week during latency and treatment period. The formula Total Volume (TV) (mm^3^) = [length (mm) x width (mm)^2^]/2 was used, where the length and the width are the longest and the shortest diameters of the tumor. The tumor growth delay index (TGDI) was calculated as the median growth delay in the treated group divided by the median growth delay in the control group, and represented the x-fold time to reach 5-fold the TV at D0.The ratio between the mean TV of a treated group (T) and the mean TV of the control group (C) was calculated at each measurement, and represented the antitumoral activity of the tested compound(s). The tumor growth rate, evaluated by the formula [DT/T_0_]-1 where DT is the delta of tumor size between the considered day and the day of enrolment and T_0_ is mean TV at D0, gives the percentage of growth compared with the initial day. When [DT/T_0_]-1<100%, mean TV has decreased, and when [DT/T_0_]-1>100%, mean TV has increased. Efficacy studies were performed by using 6 mice per group. All animals were weighed at tumor measurement time and were observed every day for physical appearance, behavior, and clinical changes.

### Statistical analysis

All statistical analysis was performed with GraphPad Prism. Statistical test used is describe in each result presented.

### DNA and RNA extraction and sequencing

Material for the generation of exome and RNA sequencing data was isolated from 6 hepatoblastoma cell lines. Each cell line was grown to 70% confluency, trypsinized, and snap frozen. Cells were subjected to RNA and DNA sequencing by Beijing Genomics Institute (Philadelphia, PA). The quality of DNA prior to extraction was adequate for each cell line (DNA fragment ≥ 250bp), as well as the quality of RNA (DV<200%). HiSeq 4000 was used for paired-end sequencing with 40 million reads for RNA and 100X coverage for tumor DNA.

### Whole-exome and RNA analysis

Raw FASTQ sequencing files were run through our computational pipeline. Mutations were called with MuTect2 and indels were called using platypus with computational filtering of artifacts. Annotation of variants was performed using Annovar. Gene expression was quantified using STAR aligner with RSEM.

### Hierarchical agglomerative clustering analysis

Hierarchical agglomerative clustering analysis was performed using RNA sequencing from hepatoblastoma patient-derived cell lines, tumor tissue from the Bordeaux Bioinformatics Center (CBIB), patient-derived xenograft (PDX) models from Champions Oncology, and tumor tissue samples from Children’s Hospital of Philadelphia (CHOP). The distance between clusters was measured using the complete-linkage clustering method in R Version 3.4.4. The dist() function and the Euclidean distance methods were used to develop a dendrogram with three major clusters. Genes which possessed a mutation/indel or were overexpressed (above median value of all tumor samples) were marked in the legend. Demographic information was also included in the legend for each sample in the dendrogram. Using the same methods as described above, unsupervised clustering was performed on the data within the legend to develop a secondary dendrogram that correlates demographic information with mutations and overexpressed genes indicated in the legend.

### Data accessibility

DNA and RNA sequencing data performed for this manuscript will be made available for access through NCBI’s database of genotypes and phenotypes (dbGaP) database. Published data used in this manuscript is available through Gene Expression Omnibus (GEO) at the following accession numbers: GSE104766, GSE89775.

### Ethics statement

All animal studies were completed with supervision from the Xentech IACUC.

## SUPPLEMENTARY MATERIALS FIGURES AND TABLES



## Abbreviations

PLK1: Polo-like kinase 1
NSCLC: Non-small cell lung carcinoma
NHL: Non-hodgkin lymphoma
AML: Acute myeloid leukemia
PDX: Patient-derived xenograft
CTNNB1: β-catenin
CI: Combination index
AUC: Area under the curve

## References

[1] Spector LG , Birch J . The epidemiology of hepatoblastoma. Pediatr Blood Cancer. 2012; 59:776–79. 10.1002/pbc.24215. 22692949

[2] Carceller A , Blanchard H , Champagne J , St-Vil D , Bensoussan AL . Surgical resection and chemotherapy improve survival rate for patients with hepatoblastoma. J Pediatr Surg. 2001; 36:755–59. 10.1053/jpsu.2001.22953. 11329582

[3] Garnier A , Ilmer M , Kappler R , Berger M . Therapeutic Innovations for Targeting Hepatoblastoma. Anticancer Res. 2016; 36:5577–92. 10.21873/anticanres.11143. 27793881

[4] Armeanu-Ebinger S , Hoh A , Wenz J , Fuchs J . Targeting EpCAM (CD326) for immunotherapy in hepatoblastoma. Oncoimmunology. 2013; 2:e22620. 10.4161/onci.22620. 23482411 PMC3583930

[5] Agnoletto C , Minotti L , Brulle-Soumare L , Pasquali L , Galasso M , Corrà F , Baldassari F , Judde JG , Cairo S , Volinia S . Heterogeneous expression of EPCAM in human circulating tumour cells from patient-derived xenografts. Biomark Res. 2018; 6:31. 10.1186/s40364-018-0145-8. 30450210 PMC6208170

[6] Jin J , Jin J , Woodfield SE , Patel RH , Jin NG , Shi Y , Liu B , Sun W , Chen X , Yu Y , Vasudevan SA . Targeting LRH‑1 in hepatoblastoma cell lines causes decreased proliferation. Oncol Rep. 2019; 41:143–53. 10.3892/or.2018.6793. 30320362 PMC6278492

[7] Stafman LL , Mruthyunjayappa S , Waters AM , Garner EF , Aye JM , Stewart JE , Yoon KJ , Whelan K , Mroczek-Musulman E , Beierle EA . Targeting PIM kinase as a therapeutic strategy in human hepatoblastoma. Oncotarget. 2018; 9:22665–79. 10.18632/oncotarget.25205. 29854306 PMC5978256

[8] Zitouni S , Nabais C , Jana SC , Guerrero A , Bettencourt-Dias M . Polo-like kinases: structural variations lead to multiple functions. Nat Rev Mol Cell Biol. 2014; 15:433–52. 10.1038/nrm3819. 24954208

[9] Golsteyn RM , Schultz SJ , Bartek J , Ziemiecki A , Ried T , Nigg EA . Cell cycle analysis and chromosomal localization of human Plk1, a putative homologue of the mitotic kinases Drosophila polo and Saccharomyces cerevisiae Cdc5. J Cell Sci. 1994; 107:1509–17. 7962193 10.1242/jcs.107.6.1509

[10] Park JE , Li L , Park J , Knecht R , Strebhardt K , Yuspa SH , Lee KS . Direct quantification of polo-like kinase 1 activity in cells and tissues using a highly sensitive and specific ELISA assay. Proc Natl Acad Sci U S A. 2009; 106:1725–30. 10.1073/pnas.0812135106. 19181852 PMC2633213

[11] Rudolph D , Impagnatiello MA , Blaukopf C , Sommer C , Gerlich DW , Roth M , Tontsch-Grunt U , Wernitznig A , Savarese F , Hofmann MH , Albrecht C , Geiselmann L , Reschke M , et al. Efficacy and mechanism of action of volasertib, a potent and selective inhibitor of Polo-like kinases, in preclinical models of acute myeloid leukemia. J Pharmacol Exp Ther. 2015; 352:579–89. 10.1124/jpet.114.221150. 25576074

[12] Hu K , Lee C , Qiu D , Fotovati A , Davies A , Abu-Ali S , Wai D , Lawlor ER , Triche TJ , Pallen CJ , Dunn SE . Small interfering RNA library screen of human kinases and phosphatases identifies polo-like kinase 1 as a promising new target for the treatment of pediatric rhabdomyosarcomas. Mol Cancer Ther. 2009; 8:3024–35. 10.1158/1535-7163.MCT-09-0365. 19887553 PMC2783569

[13] Yamada S , Ohira M , Horie H , Ando K , Takayasu H , Suzuki Y , Sugano S , Hirata T , Goto T , Matsunaga T , Hiyama E , Hayashi Y , Ando H , et al. Expression profiling and differential screening between hepatoblastomas and the corresponding normal livers: identification of high expression of the PLK1 oncogene as a poor-prognostic indicator of hepatoblastomas. Oncogene. 2004; 23:5901–11. 10.1038/sj.onc.1207782. 15221005

[14] Liu X . Targeting Polo-Like Kinases: A Promising Therapeutic Approach for Cancer Treatment. Transl Oncol. 2015; 8:185–95. 10.1016/j.tranon.2015.03.010. 26055176 PMC4486469

[15] Gjertsen BT , Schöffski P . Discovery and development of the Polo-like kinase inhibitor volasertib in cancer therapy. Leukemia. 2015; 29:11–19. 10.1038/leu.2014.222. 25027517 PMC4335352

[16] Rudolph D , Steegmaier M , Hoffmann M , Grauert M , Baum A , Quant J , Haslinger C , Garin-Chesa P , Adolf GR . BI 6727, a Polo-like kinase inhibitor with improved pharmacokinetic profile and broad antitumor activity. Clin Cancer Res. 2009; 15:3094–102. 10.1158/1078-0432.CCR-08-2445. 19383823

[17] Gutteridge RE , Ndiaye MA , Liu X , Ahmad N . Plk1 Inhibitors in Cancer Therapy: From Laboratory to Clinics. Mol Cancer Ther. 2016; 15:1427–35. 10.1158/1535-7163.MCT-15-0897. 27330107 PMC4936921

[18] Zsíros J , Brugières L , Brock P , Roebuck D , Maibach R , Child M , Morland B , Casanova M , Pariente D , Paris C , de Camargo B , Ronghe M , Zimmermann A , et al. Efficacy of irinotecan single drug treatment in children with refractory or recurrent hepatoblastoma—a phase II trial of the childhood liver tumour strategy group (SIOPEL). Eur J Cancer. 2012; 48:3456–64. 10.1016/j.ejca.2012.06.023. 22835780

[19] Nicolle D , Fabre M , Simon-Coma M , Gorse A , Kappler R , Nonell L , Mallo M , Haidar H , Déas O , Mussini C , Guettier C , Redon MJ , Brugières L , et al. Patient-derived mouse xenografts from pediatric liver cancer predict tumor recurrence and advise clinical management. Hepatology. 2016; 64:1121–35. 10.1002/hep.28621. 27115099

[20] Andersen TB , López CQ , Manczak T , Martinez K , Simonsen HT . Thapsigargin--from Thapsia L. to mipsagargin. Molecules. 2015; 20:6113–27. 10.3390/molecules20046113. 25856061 PMC6272310

[21] Hooks KB , Audoux J , Fazli H , Lesjean S , Ernault T , Dugot-Senant N , Leste-Lasserre T , Hagedorn M , Rousseau B , Danet C , Branchereau S , Brugières L , Taque S , et al. New insights into diagnosis and therapeutic options for proliferative hepatoblastoma. Hepatology. 2018; 68:89–102. 10.1002/hep.29672. 29152775

[22] Cairo S , Armengol C , De Reyniès A , Wei Y , Thomas E , Renard CA , Goga A , Balakrishnan A , Semeraro M , Gresh L , Pontoglio M , Strick-Marchand H , Levillayer F , et al. Hepatic stem-like phenotype and interplay of Wnt/beta-catenin and Myc signaling in aggressive childhood liver cancer. Cancer Cell. 2008; 14:471–84. 10.1016/j.ccr.2008.11.002. 19061838

[23] Ranganathan S , Ningappa M , Ashokkumar C , Higgs BW , Min J , Sun Q , Schmitt L , Subramaniam S , Hakonarson H , Sindhi R . Loss of EGFR-ASAP1 signaling in metastatic and unresectable hepatoblastoma. Sci Rep. 2016; 6:38347. 10.1038/srep38347. 27910913 PMC5133573

[24] López-Terrada D , Cheung SW , Finegold MJ , Knowles BB . Hep G2 is a hepatoblastoma-derived cell line. Hum Pathol. 2009; 40:1512–15. 10.1016/j.humpath.2009.07.003. 19751877

[25] McCarthy DJ , Chen Y , Smyth GK . Differential expression analysis of multifactor RNA-Seq experiments with respect to biological variation. Nucleic Acids Res. 2012; 40:4288–97. 10.1093/nar/gks042. 22287627 PMC3378882

[26] Sumazin P , Chen Y , Treviño LR , Sarabia SF , Hampton OA , Patel K , Mistretta TA , Zorman B , Thompson P , Heczey A , Comerford S , Wheeler DA , Chintagumpala M , et al. Genomic analysis of hepatoblastoma identifies distinct molecular and prognostic subgroups. Hepatology. 2017; 65:104–21. 10.1002/hep.28888. 27775819

[27] Zheng DW , Xue YQ , Li Y , Di JM , Qiu JG , Zhang WJ , Jiang QW , Yang Y , Chen Y , Wei MN , Huang JR , Wang K , Wei X , Shi Z . Volasertib suppresses the growth of human hepatocellular carcinoma *in vitro* and *in vivo* . Am J Cancer Res. 2016; 6:2476–88. 27904765 PMC5126267

[28] Raab M , Krämer A , Hehlgans S , Sanhaji M , Kurunci-Csacsko E , Dötsch C , Bug G , Ottmann O , Becker S , Pachl F , Kuster B , Strebhardt K . Mitotic arrest and slippage induced by pharmacological inhibition of Polo-like kinase 1. Mol Oncol. 2015; 9:140–54. 10.1016/j.molonc.2014.07.020. 25169932 PMC5528686

[29] Lin CC , Su WC , Yen CJ , Hsu CH , Su WP , Yeh KH , Lu YS , Cheng AL , Huang DC , Fritsch H , Voss F , Taube T , Yang JC . A phase I study of two dosing schedules of volasertib (BI 6727), an intravenous polo-like kinase inhibitor, in patients with advanced solid malignancies. Br J Cancer. 2014; 110:2434–40. 10.1038/bjc.2014.195. 24755882 PMC4021529

[30] Rothenberg ML , Kuhn JG , Schaaf LJ , Rodriguez GI , Eckhardt SG , Villalona-Calero MA , Rinaldi DA , Hammond LA , Hodges S , Sharma A , Elfring GL , Petit RG , Locker PK , et al. Phase I dose-finding and pharmacokinetic trial of irinotecan (CPT-11) administered every two weeks. Ann Oncol. 2001; 12:1631–41. 10.1023/A:1013157727506. 11822765

[31] Awada A , Dumez H , Aftimos PG , Costermans J , Bartholomeus S , Forceville K , Berghmans T , Meeus MA , Cescutti J , Munzert G , Pilz K , Liu D , Schöffski P . Phase I trial of volasertib, a Polo-like kinase inhibitor, plus platinum agents in solid tumors: safety, pharmacokinetics and activity. Invest New Drugs. 2015; 33:611–20. 10.1007/s10637-015-0223-9. 25794535 PMC4435638

[32] Kobayashi Y , Yamauchi T , Kiyoi H , Sakura T , Hata T , Ando K , Watabe A , Harada A , Taube T , Miyazaki Y , Naoe T . Phase I trial of volasertib, a Polo-like kinase inhibitor, in Japanese patients with acute myeloid leukemia. Cancer Sci. 2015; 106:1590–95. 10.1111/cas.12814. 26471242 PMC4714695

[33] Boehringer Ingelheim. ID NCT01971476. Open Dose Escalating Trial to Determine the Maximum Tolerated Dose in Paediatric Patients With Advanced Cancers for Whom no Therapy is Known. Available from: https://clinicaltrials.gov/ct2/show/NCT01971476.

[34] Adachi Y , Ishikawa Y , Kiyoi H . Identification of volasertib-resistant mechanism and evaluation of combination effects with volasertib and other agents on acute myeloid leukemia. Oncotarget. 2017; 8:78452–65. 10.18632/oncotarget.19632. 29108241 PMC5667974

[35] Bomgaars LR , Bernstein M , Krailo M , Kadota R , Das S , Chen Z , Adamson PC , Blaney SM . Phase II trial of irinotecan in children with refractory solid tumors: a Children’s Oncology Group Study. J Clin Oncol. 2007; 25:4622–27. 10.1200/JCO.2007.11.6103. 17925558

[36] Zhang YT , Feng LH , Zhong XD , Wang LZ , Chang J . Vincristine and irinotecan in children with relapsed hepatoblastoma: a single-institution experience. Pediatr Hematol Oncol. 2015; 32:18–25. 10.3109/08880018.2014.909913. 24852330

[37] Yan Z , Zhu ZL , Qian ZZ , Hu G , Wang HQ , Liu WH , Cheng G . Pharmacokinetic characteristics of vincristine sulfate liposomes in patients with advanced solid tumors. Acta Pharmacol Sin. 2012; 33:852–58. 10.1038/aps.2012.44. 22669119 PMC4010381

[38] Dong J , Park SY , Nguyen N , Ezhilarasan R , Martinez-Ledesma E , Wu S , Henry V , Piao Y , Tiao N , Brunell D , Stephan C , Verhaak R , Sulman E , et al. The polo-like kinase 1 inhibitor volasertib synergistically increases radiation efficacy in glioma stem cells. Oncotarget. 2018; 9:10497–509. 10.18632/oncotarget.24041. 29535822 PMC5828226

[39] Han Y , Lindner S , Bei Y , Garcia HD , Timme N , Althoff K , Odersky A , Schramm A , Lissat A , Künkele A , Deubzer HE , Eggert A , Schulte JH , Henssen AG . Synergistic activity of BET inhibitor MK-8628 and PLK inhibitor Volasertib in preclinical models of medulloblastoma. Cancer Lett. 2019; 445:24–33. 10.1016/j.canlet.2018.12.012. 30611741

[40] Pommier Y . Topoisomerase I inhibitors: camptothecins and beyond. Nat Rev Cancer. 2006; 6:789–802. 10.1038/nrc1977. 16990856

[41] Capranico G , Marinello J , Baranello L . Dissecting the transcriptional functions of human DNA topoisomerase I by selective inhibitors: implications for physiological and therapeutic modulation of enzyme activity. Biochim Biophys Acta. 2010; 1806:240–50. 10.1016/j.bbcan.2010.06.003. 20600630

[42] Tomicic MT , Kaina B . Topoisomerase degradation, DSB repair, p53 and IAPs in cancer cell resistance to camptothecin-like topoisomerase I inhibitors. Biochim Biophys Acta. 2013; 1835:11–27. 10.1016/j.bbcan.2012.09.002. 23006513

[43] Khaderi S , Guiteau J , Cotton RT , O’Mahony C , Rana A , Goss JA . Role of liver transplantation in the management of hepatoblastoma in the pediatric population. World J Transplant. 2014; 4:294–98. 10.5500/wjt.v4.i4.294. 25540737 PMC4274598

[44] Sivaprakasam P , Gupta AA , Greenberg ML , Capra M , Nathan PC . Survival and long-term outcomes in children with hepatoblastoma treated with continuous infusion of cisplatin and doxorubicin. J Pediatr Hematol Oncol. 2011; 33:e226–30. 10.1097/MPH.0b013e31821f0eaf. 21792028

[45] Gaffan J , Holden L , Newlands ES , Short D , Fuller S , Begent RH , Rustin GJ , Seckl MJ . Infertility rates following POMB/ACE chemotherapy for male and female germ cell tumours - a retrospective long-term follow-up study. Br J Cancer. 2003; 89:1849–54. 10.1038/sj.bjc.6601383. 14612891 PMC2394462

[46] Lipshultz SE , Miller TL , Scully RE , Lipsitz SR , Rifai N , Silverman LB , Colan SD , Neuberg DS , Dahlberg SE , Henkel JM , Asselin BL , Athale UH , Clavell LA , et al. Changes in cardiac biomarkers during doxorubicin treatment of pediatric patients with high-risk acute lymphoblastic leukemia: associations with long-term echocardiographic outcomes. J Clin Oncol. 2012; 30:1042–49. 10.1200/JCO.2010.30.3404. 22370326 PMC3341148

[47] Hao Z , Kota V . Volasertib for AML: clinical use and patient consideration. Onco Targets Ther. 2015; 8:1761–71. 10.2147/OTT.S60762. 26229484 PMC4514349

[48] Zuco V , De Cesare M , Zaffaroni N , Lanzi C , Cassinelli G . PLK1 is a critical determinant of tumor cell sensitivity to CPT11 and its inhibition enhances the drug antitumor efficacy in squamous cell carcinoma models sensitive and resistant to camptothecins. Oncotarget. 2015; 6:8736–49. 10.18632/oncotarget.3538. 25826089 PMC4496180

