# Supplementary material for: Volasertib preclinical activity in high-risk hepatoblastoma
Source: Oncotarget. 2019 Nov 5;10(60):6403–17. doi: 10.18632/oncotarget.27237 (PMC6849653; doi:10.18632/oncotarget.27237)
Supplement: Supplementary file 1 [file oncotarget-10-6403-s001.pdf]

Volasertib preclinical activity in high-risk hepatoblastoma

SUPPLEMENTARY MATERIALS

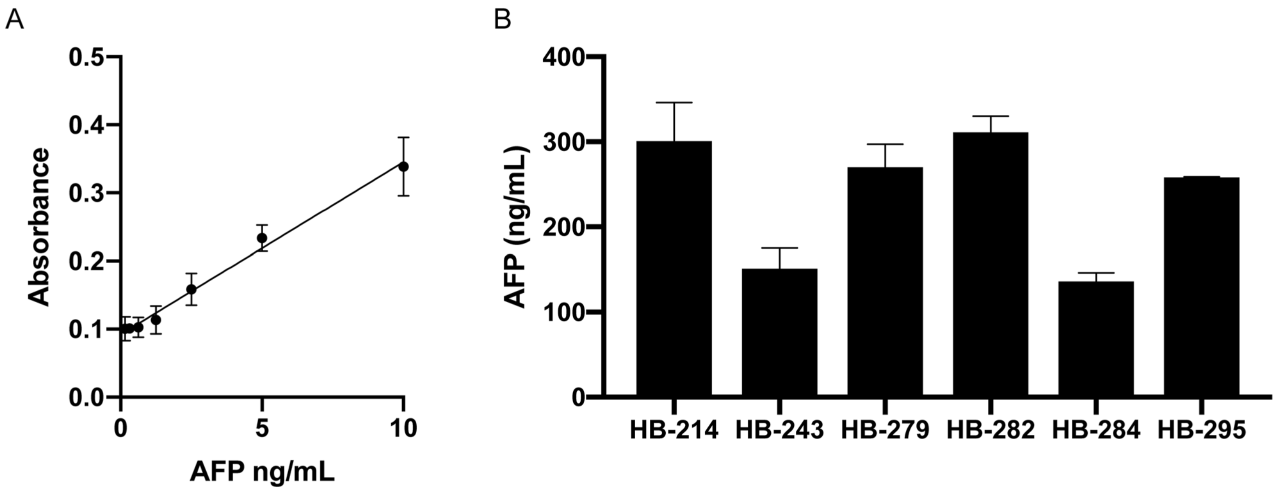

Supplementary Figure 1: Alphafetoprotein secretion (AFP) by hepatoblastoma cell lines. (A) Standard curve for AFP ELISA. **(B)** Measured AFP secretion from hepatoblastoma cell lines after five days of culture. Data represented as mean +/− SD. N=3.

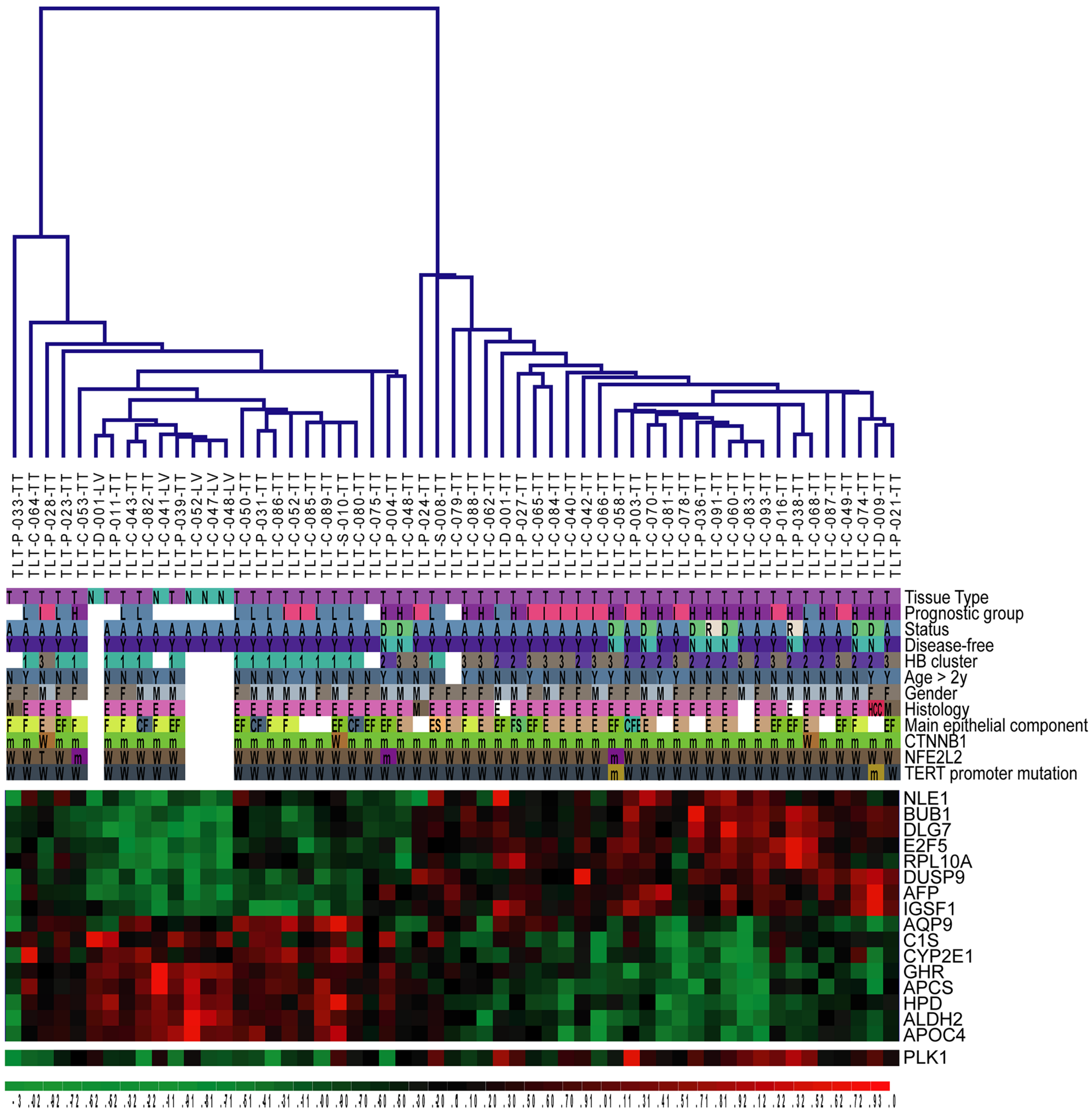

Supplementary Figure 2: Hierarchical clustering of 50 HBs and five normal liver samples. (labeled in cyan) from Sumazin *et al* [1] by using the 16-gene signature (Tissue type: T=tumor, N=non-tumor; Prognostic group: L=low, I=intermediate, H=high; Stetus:A=alive; D=dead of disease; R=relapse; HB cluster: 1,2 or 3 according to Sumazin et al36; Age >2-years old: Y=yes, N=no; Gender: M=male; F=female; Histology: E=epithelial; M=mixed; HCC=hepatocellular carcinoma; Main epithelial component: F=fetal, CF=crowded fetal, E=embryonal, S=small cells undifferentiated; CTNNB1, NFE2L2 and TERT promoter: W=wild-type; m=mutated).

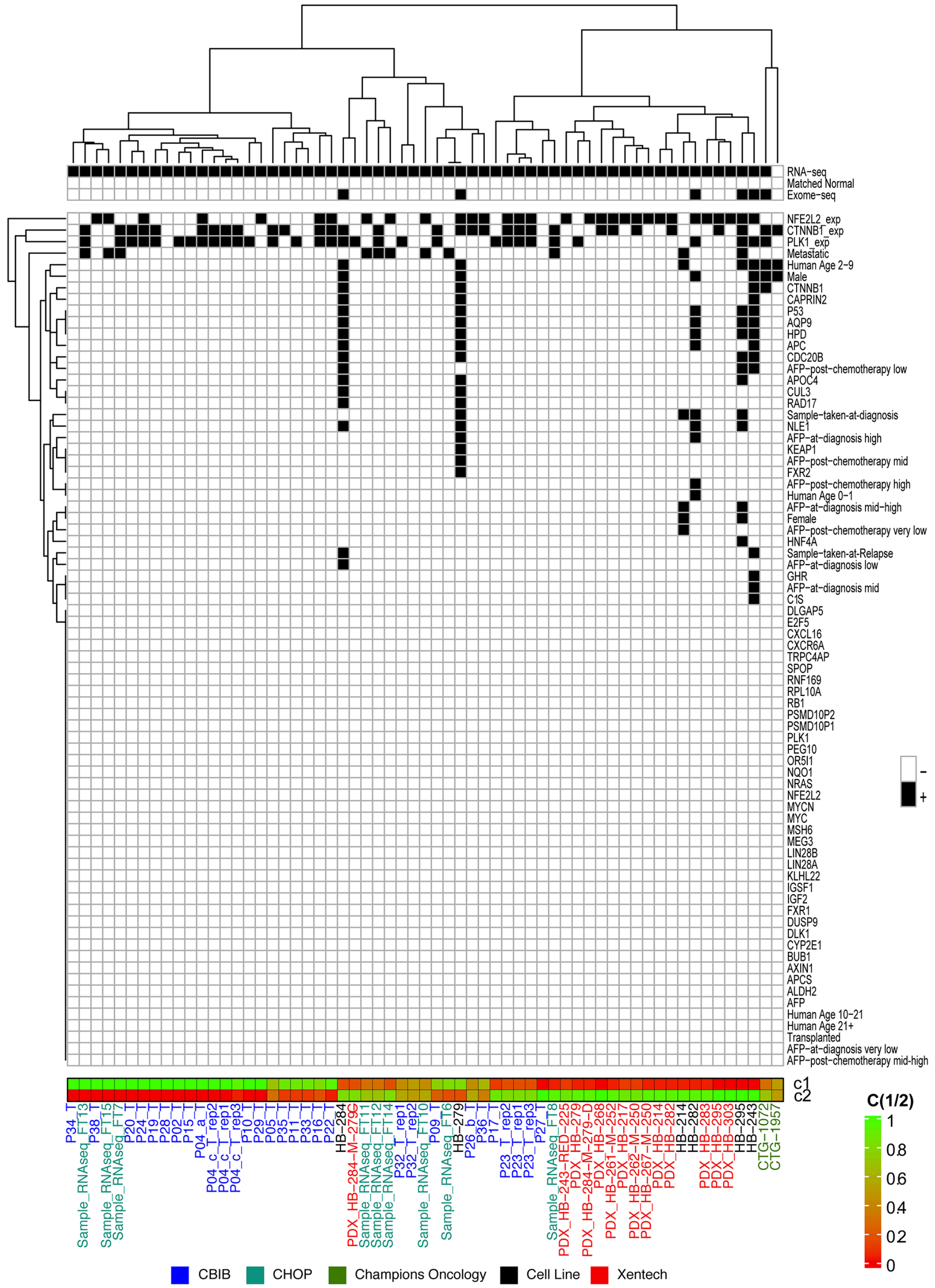

Supplementary Figure 3: Hepatoblastoma dendrogram and legend for a greater number of covariates than Figure 3. Unsupervised clustering of hepatoblastoma samples using RNA-seq expression data, the pre-defined 16-gene signature, and genes identified in hepatoblastoma by Eichenmüller et al. [2], Bissig-Choisat et al. [3], and Jia et al [4]. Samples with somatic mutations in each gene are noted in the legend along with samples that have overexpression of CTNNB1, NFE2L2, and/or PLK1. AFP values are indicated as follows: AFP high is in the range of 1,000,000 – 10,000,000, AFP mid-high is between 100,000 and 999,999, AFP mid is between 10,000 and 99,999, AFP mid-low is between 1,000 and 9,999 and AFP low indicates a value between 0 and 999. Exp, gene expression. Gene names reflect DNA-based mutation (where data is available).

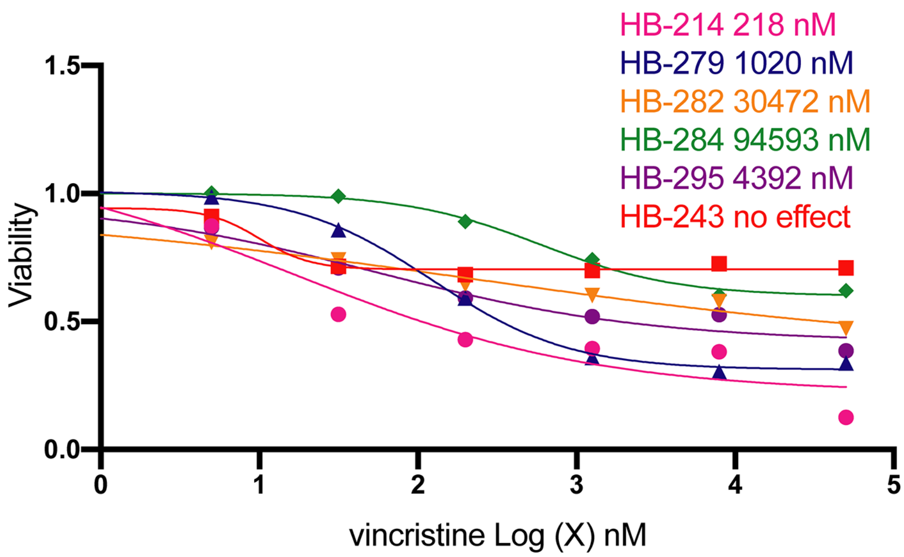

Supplementary Figure 4: IC50 values for vincristine across hepatoblastoma cell lines. Cell viability was measured after 72hr drug exposure. Values are an average of quadruplicates, Data is represented as mean+/− standard deviation.

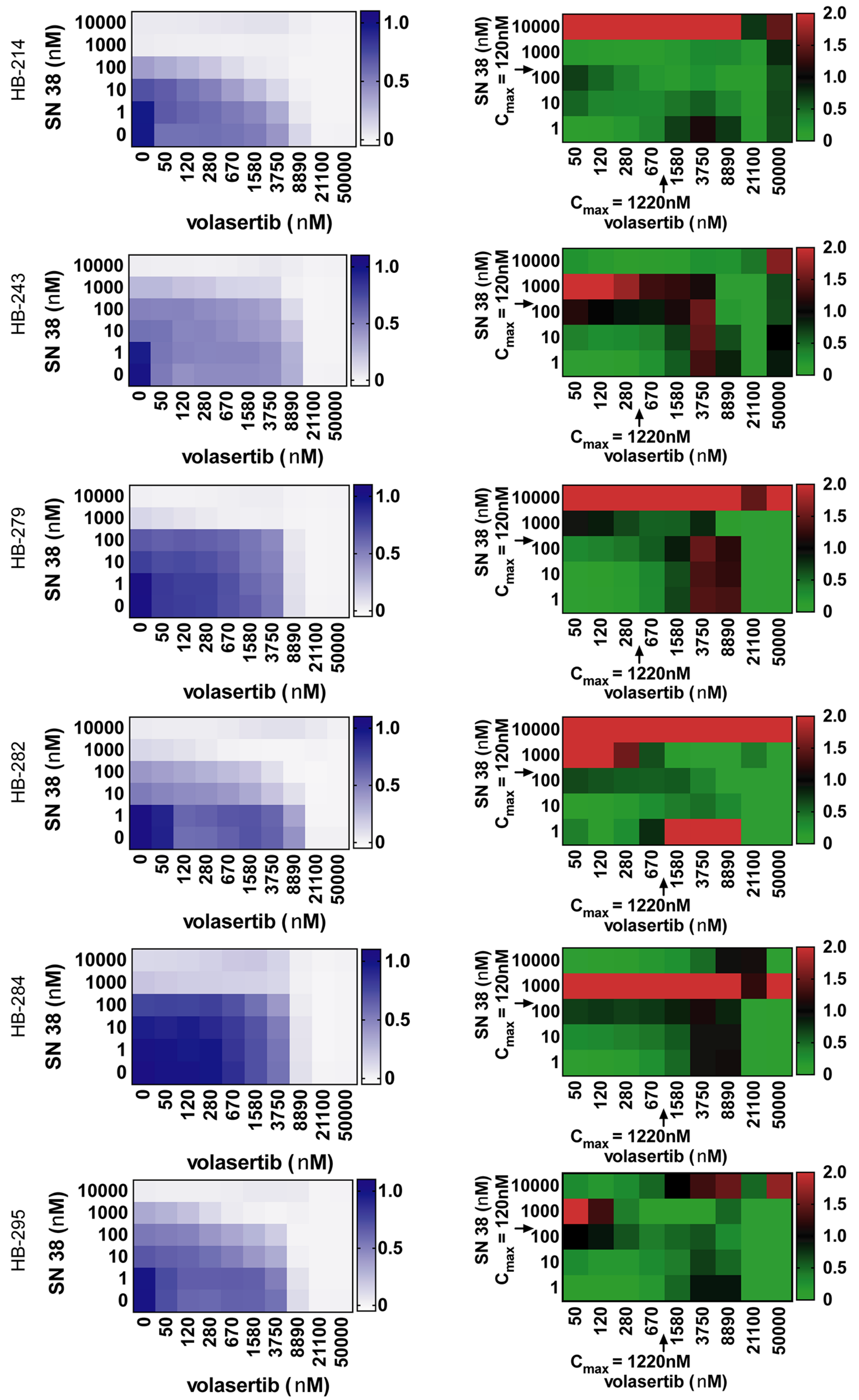

Supplementary Figure 5: Volasertib and SN38 response heatmaps and combination index calculations for six hepatoblastoma cell lines. Left - Cell proliferation in response to 72-hour drug treatment. Right - Combination index of volasertib and SN38 drug treatments. N = 4.

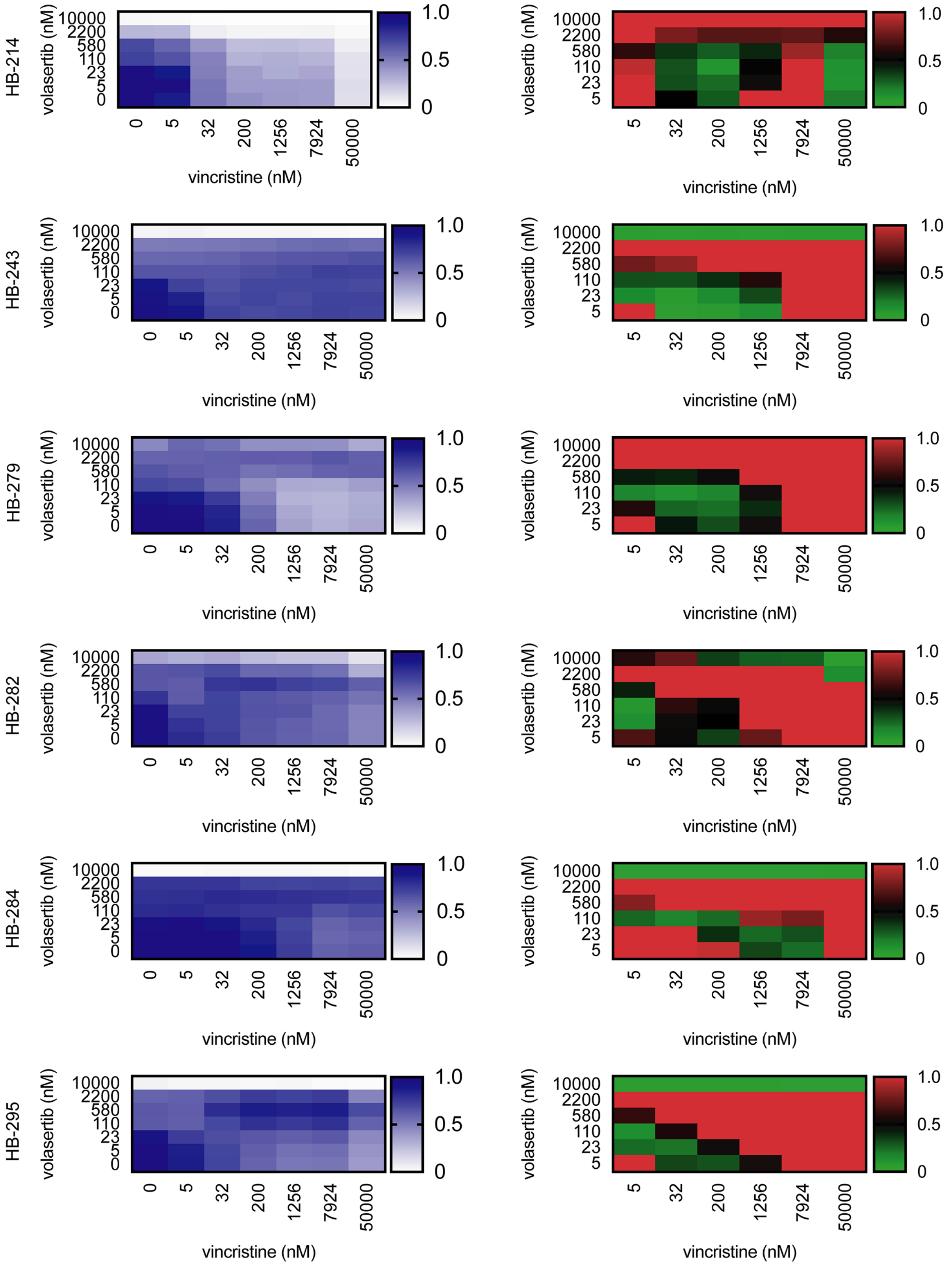

Supplementary Figure 6: Volasertib and vincristine response heatmaps and combination index heatmaps for six hepatoblastoma cell lines. Left - Cell proliferation in response to 72-hour drug treatment. Right - Combination index of volasertib and SN38 drug treatments. N = 4.

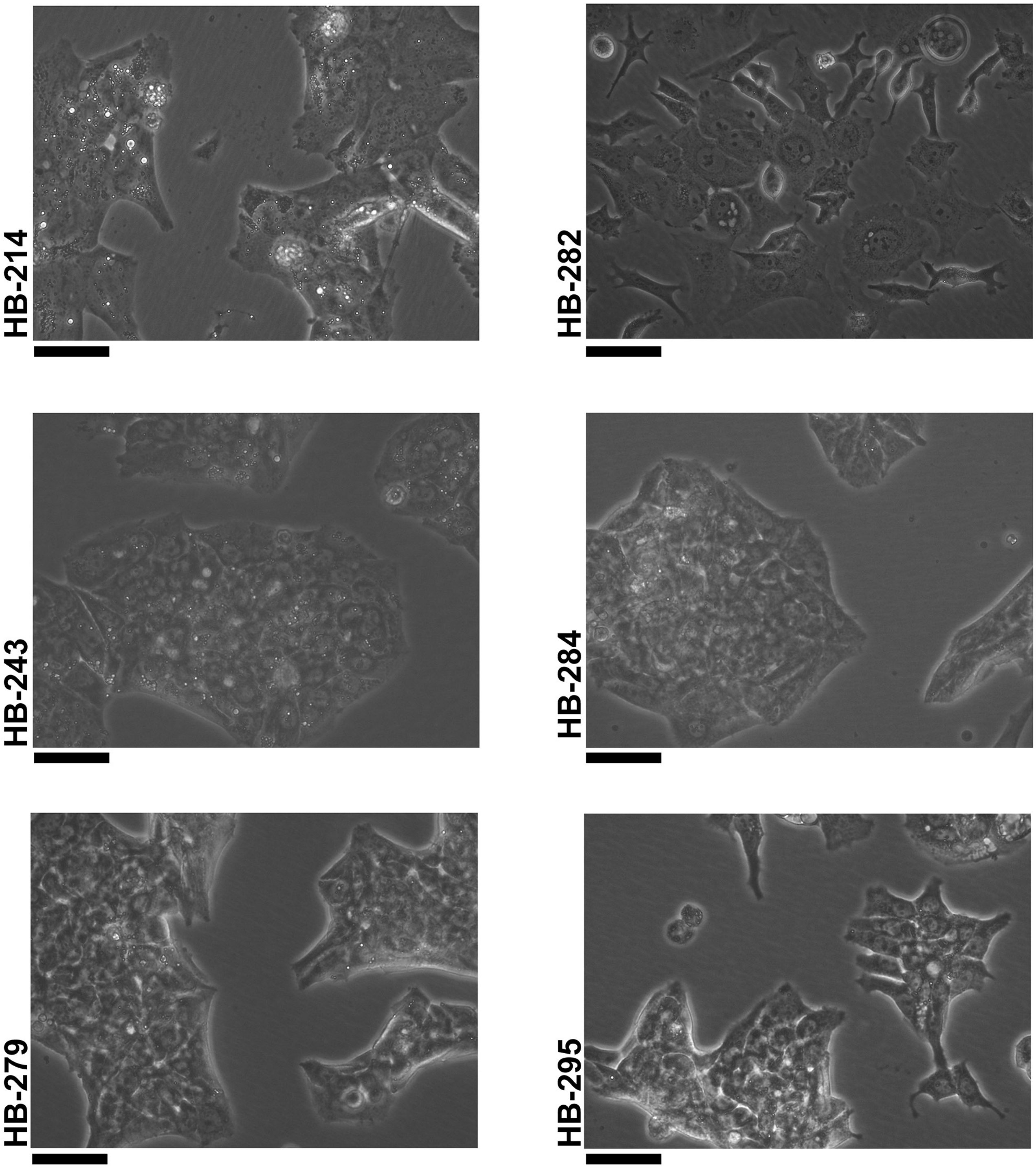

Supplementary Figure 7: Hepatoblastoma cell morphology. Characteristic cell morphology is shown Scale bar = 40 μM.

Supplementary Table 1: STR profiles of cell lines and original PDX models

	Name	TH01	D5S818	D13S317	D7S820	D16S539	CSF1PO	Amelogenin	vWA	TPOX
liver pdx	HB-214	6, 7	9, 12	10, 13	9, 12	11, 11	9, 12	X, X	14, 17	8, 8
	HB-243-BUI-RED-225	9.3, 9.3	11, 13	8, 11	11, 11	9, 13	10, 11	X, Y	17, 18	8, 8
	HB-279	7,9.3	11,11	12,12	10,10	10,12	11,11	X,Y	16,16	8,9
	HB-282	9.3,9.3	11,11	8,14	9,10	11,13	10,10	X,Y	17,19	8,11
	HB-284-M-279-D	7,9.3	11,11	12,12	10,10	10,12	10,10	X,Y	16,16	8,8
	HB-284-M-279-C	7, 9.3	11, 11	12, 12	10, 10	10, 10	11, 11	X, Y	16, 16	8, 9
**PDX-derived cell lines**	HB-214	6, 7	9, 12	10, 13	9, 12	11, 11	9, 11	X, X	14, 17	8, 8
	HB-243-BUI-RED-225	9.3, 9.3	11, 13	8, 11	11, 11	9, 13	10, 11	X, Y	17, 18	8, 8
	HB-279	7, 9.3	11, 11	12, 12	10, 10	12, 12	11, 11	X, Y	16, 16	8, 9
	HB-282	9.3, 9.3	11, 11	8, 14	9, 10	11, 13	10, 10	X, Y	17, 19	8, 11
	HB-284-279-D	7, 9.3	11, 11	12, 12	10, 10	10, 12	11, 11	X, Y	16, 16	8, 9
	HB-295	6, 9.3	11, 13	8, 12	10, 12	11, 11	9, 12	X, X	14, 16	8, 11

Supplementary Table 2: Clinical information of cell lines

**PDX ID**	**age (months)**	**type of sample**	**R[1] or LT[2]**	**sex**	**vascular invasion**	**solitary/ multiple nodules**	**metastasis**	**main cellular component**	**PRETEXT[3] stage**	**Protocol**	**AFP serum at diagnosis (ng/mL)**	**AFP serum post-chemoth. (ng/mL)**
HB-214	31	Primary	R	F	Y	M	Y	fetal	II	SIOPEL3	700,000	367
HB-243	52	Intrahepatic relapse	LT	M	Y	M	N	embryonal		CARBO[4]+ VEPESIDE (ETOPOSIDE)	6,000	5,000
HB-279	79	Primary	LT	M	Y	M	N	embryonal+ macrotrabecular	IV	SIOPEL4	1,000,000	30,000
HB-282	12	Primary	R	M	N	S	N	embryonal	II	SIOPEL6+3	1,286,980	1,000,000
HB-284	83	Peritoneal metastasis at relapse	R	M				embryonal		ETOPOSIDE+ CISPLATIN	2,162	1,089
HB-295	26	Primary	R	F	Y	M	Y	fetal	II	SIOPEL4	585,350	1,400

[1]R = Resection
[2]LT = Liver Transplant
[3]PRE-Treatment EXTent of tumor (PRETEXT) is the staging and risk stratification system developed by the International Childhood Liver Tumor Strategy Group for hepatoblastoma. A higher PRETEXT value indicates that more lobes of the liver are involved in the tumor and is therefore higher risk.
[4]Carbo refers to carboplatin. Vepeside is an alternative name for etoposide.
Empty boxes are unknown.

Supplementary Table 3: *In vivo* experimental design

Group	N	agent	dose	route of administration	schedule
1	6	control	N/A	N/A	N/A
2	6	volasertib	30 mg/kg	IV	2qwk[1]
3	6	irinotecan	10 mg/kg	IP	q5d[2]
4	6	volasertib + irinotecan	30 mg/kg + 10 mg/kg	IV + IP	2qwk + q5d

[1]2qwk, twice per week.
[2]q5d, every five days.

REFERENCES

1. Sumazin P, Chen Y, Treviño LR, Sarabia SF, Hampton OA, Patel K, Mistretta TA, Zorman B, Thompson P, Heczey A, Comerford S, Wheeler DA, Chintagumpala M, et al. Genomic analysis of hepatoblastoma identifies distinct molecular and prognostic subgroups. Hepatology. 2017; 65:104–21. https://doi.org/10.1002/hep.28888. [PubMed]
2. Eichenmüller M, Trippel F, Kreuder M, Beck A, Schwarzmayr T, Häberle B, Cairo S, Leuschner I, von Schweinitz D, Strom TM, Kappler R. The genomic landscape of hepatoblastoma and their progenies with HCC-like features. J Hepatol. 2014; 61:1312–20. https://doi.org/10.1016/j.jhep.2014.08.009. [PubMed]
3. Bissig-Choisat B, Kettlun-Leyton C, Legras XD, Zorman B, Barzi M, Chen LL, Amin MD, Huang YH, Pautler RG, Hampton OA, Prakash MM, Yang D, Borowiak M, et al. Novel patient-derived xenograft and cell line models for therapeutic testing of pediatric liver cancer. J Hepatol. 2016; 65:325–33. https://doi.org/10.1016/j.jhep.2016.04.009. [PubMed]
4. Jia D, Dong R, Jing Y, Xu D, Wang Q, Chen L, Li Q, Huang Y, Zhang Y, Zhang Z, Liu L, Zheng S, Xia Q, et al. Exome sequencing of hepatoblastoma reveals novel mutations and cancer genes in the Wnt pathway and ubiquitin ligase complex. Hepatology. 2014; 60:1686–96. https://doi.org/10.1002/hep.27243. [PubMed]
